# Our Periodicals

**Published:** 1836

**Authors:** 


### Art. II.—Our Periodical.

Doctor Drake, the senior editor, of this Journal, is now on a tour of medical observation through some of the southern states, and hence the arrangement of the principal part of this number has devolved on the assistant editor. We hope, however, that the pages will be found both interesting and useful, and that whatever they have lost from the absence of the able pen of Dr. Drake, they have gained from the articles of our correspondents.

Our subscribers will perceive that this number has been issued in due time, and as the publishers are determined to be punctual for the future, we hope our friends will not be disappointed by unnecessary delay. A part of all that the editor may collect for his proposed work on the diseases of our great valley, will be laid before our readers in the Journal as it issues from the press, and hence we hope the tenth volume will be even more useful than those which have preceded it. Notwithstanding the many changes that have been made in its publishers the subscription list continues to increase so rapidly that it will be almost doubled at the close of this volume, should nothing occur to arrest or retard its progress. We hope our subscribers will contribute much to its success, by reporting whatever is interesting in their practice. We return our thanks to those who have lent us their aid in the last volumes and hope they will continue their favors. Several favors came to hand too late for this number which will appear in the next. W. W.

